# Transcriptome analysis of *Gossypium* reveals the molecular mechanisms of Ca^2+^ signaling pathway on arsenic tolerance induced by arbuscular mycorrhizal fungi

**DOI:** 10.3389/fmicb.2024.1362296

**Published:** 2024-03-25

**Authors:** Minggui Gong, Na Bai, Jiajie Su, Yuan Wang, Yanan Wei, Qiaoming Zhang

**Affiliations:** ^1^College of Food and Bioengineering, Henan University of Science and Technology, Luoyang, China; ^2^College of Horticulture and Plant Protection, Henan University of Science and Technology, Luoyang, China

**Keywords:** cotton seedling, differentially expressed genes, *Funneliformis mosseae*, arsenic stress, RNA-Seq

## Abstract

**Introduction:**

Arbuscular mycorrhizal fungi (AMF) have been demonstrated their ability to enhance the arsenic (As) tolerance of host plants, and making the utilization of mycorrhizal plants a promising and practical approach for remediating As-contaminated soils. However, comprehensive transcriptome analysis to reveal the molecular mechanism of As tolerance in the symbiotic process between AMF and host plants is still limited.

**Methods:**

In this study, transcriptomic analysis of *Gossypium* seedlings was conducted with four treatments: non-inoculated *Gossypium* under non-As stress (CK0), non-inoculated *Gossypium* under As stress (CK100), *F. mosseae*-inoculated *Gossypium* under non-As stress (FM0), and *F. mosseae*-inoculated *Gossypium* under As stress (FM100).

**Results:**

Our results showed that inoculation with *F. mosseae* led to a reduction in net fluxes of Ca^2+^, while increasing Ca^2+^ contents in the roots and leaves of *Gossypium* under the same As level in soil. Notably, 199 and 3129 differentially expressed genes (DEGs) were specially regulated by *F. mosseae* inoculation under As stress and non-As stress, respectively. Through Gene Ontology (GO) and Kyoto Encyclopedia of Genes and Genomes (KEGG) annotation and enrichment analyses, we found that under As stress, *F. mosseae* inoculation up-regulated a significant number of genes related to the Ca^2+^ signaling pathway genes, involved in cellular process, membrane part, and signal transduction. This suggests a potential role in mitigating As tolerance in *Gossypium* seedlings. Furthermore, our analysis identified specific DEGs in transcription factor families, including ERF, MYB, NAC, and WRKY, that were upregulated by *F. mosseae* inoculation. Conversely, MYB and HB-other were down-regulated. The ERF and MYB families exhibited the highest number of up- and down-regulated DEGs, respectively, which were speculated to play an important role in alleviating the As toxicity of *Gossypium*.

**Discussion:**

Our findings provided valuable insights into the molecular theoretical basis of the Ca^2+^ signaling pathway in improving As tolerance of mycorrhizal plants in the future.

## 1 Introduction

Arsenic (As), a hazardous metalloid element in the earth’s crust, poses a significant environmental threat worldwide due to its widespread contamination in groundwater and soil. This contamination has garnered increasing attention regarding the global security of agricultural products (Li et al., 2018a, 2020). As also enriched in orpiment, realgar, and other ores in nature (Sharma et al., 2020). Over the past century, industrialization and anthropogenic activities, such as mining As ores, using As-based agrochemicals and fertilizer, smelting metal and glass, irrigating As-contaminated groundwater, and burning fossil fuels, have exacerbated As contamination (Chen et al., 2007; Salim et al., 2009; Srivastava et al., 2009). The entry of As and its inorganic compounds into the human food chain can lead to serious health issues, including cancers, cardiovascular diseases, diabetes, compromised immunity, and infertility (Sharma et al., 2020; Liu et al., 2021). In plants, high concentrations of As interfere with metabolic processes, leading to physiological and morphological disorders (Spagnoletti et al., 2016; Sharma et al., 2017; Martínez-Castillo et al., 2022). The symptoms of As phytotoxicity include the damage of cell structure and function, inhibition of photosynthesis and respiration, retardation of germination and growth, reduction of production, and even senescence and death, which ultimately cause vegetation degradation in the As-polluted areas (Spagnoletti et al., 2017; Surbhi et al., 2017). Therefore, some measures of biotechnological remediation need to be applied to prevent environmental deterioration due to As contamination (Martínez-Castillo et al., 2022).

To adapt to heavy metal (HM)-polluted environment, plants have evolved the intrinsic strategies of resistance to HM stress, which involves calcium (Ca) signaling responses (Sharma et al., 2020). As ubiquitous intracellular second messengers, Ca^2+^ ions regulate diverse cellular processes, which convey a wide range of environmental and developmental stimuli (Lecourieux et al., 2006; Kurusu et al., 2010). Changes in cytosolic-free Ca^2+^ concentration, which is tightly regulated by active transport (Ca^2+^−antiporters and Ca^2+^-ATPases) and passive fluxes (Ca^2+^ channels) across membranes, couple a series of signal responses in plants (Bush, 1995; Sanders et al., 1999). Under As stress, plants experience an increase in cytoplasmic Ca^2+^ concentrations, relying on transcriptional changes and the kinase activity of Ca^2+^ sensors (Sharma et al., 2020). Differential gene expression of Ca^2+^ sensors suggests their involvement of Ca^2+^ −dependent signaling responses to As toxicity (Chakrabarty et al., 2009). However, there is no direct experimental evidence supporting a relationship between As stress and Ca^2+^ signal transduction pathways.

Arbuscular mycorrhizal fungi (AMF), belonging to the phylum Glomeromycota, are recognized as phytobeneficial obligate symbiotic fungi (Schüßler and Walker, 2010). These rhizosphere endophytes form mutualistic relationships with over 80% of terrestrial higher plants, representing the most widespread beneficial plant–microbe interactions through a complex and coordinated network (Smith and Read, 2008). AMF acquire photoassimilates from plant roots, and, in return, transport essential mineral nutrients (potassium (K), phosphorus (P), nitrogen (N), etc.) from rhizosphere soil to plant roots through their arbuscules and hyphal coils (Riley and Corradi, 2013; Song et al., 2014). Mycorrhizal hyphae attach to the root surface, infiltrate into the epidermis, and further spread into inner cortical cells to develop a highly branched arbuscular structure, where more efficient uptake of water and nutrients takes place (Handa et al., 2015). Additionally, AMF colonization improves plant resistance against various biotic and abiotic stresses, including As stress (Smith and Read, 2008). In recent years, the As tolerance mechanism of host plants enhanced by AMF symbiosis was gradually investigated (Gonzalez-Chavez et al., 2002; Zhang et al., 2020, 2022). The widely accepted mechanism is “growth dilution effect”; phosphorus (P) element, sharing chemical properties with As, is acquired more P nutrition for host plants via their hyphae, leading to increased plant biomass and reduced As concentration in plant tissues (Spagnoletti and Lavado, 2015; Li et al., 2018a). Furthermore, AMF deposit As ions in their cell walls, detoxify As ions outside their hyphae by sequestering siderophores, and export them more efficiently than plant cells (Ouziad et al., 2005).

*Hymenoscyphus ericae*, a species of ericoid mycorrhizal fungi, improved the As resistance of *Calluna vulgaris* by reducing cellular arsenate to arsenite, effluxing arsenite from cells, and assimilating phosphate (Sharples et al., 2000). Similarly, two strains of arbuscular mycorrhizal fungi (AMF), *Funneliformis mosseae* and *Glomus caledonium,* inhibited high-affinity arsenate/phosphate transfer into *Holcus lanatus* roots and decreased the As phytotoxicity of host plant (Gonzalez-Chavez et al., 2002)*. Rhizophagus intraradices* alleviated the As toxicity of *Robinia pseudoacacia* by improving plant growth, root morphology, phytohormones, and the concentration of soil glomal (Zhang et al., 2020). Furthermore, inoculation with *R. intraradices* enhanced the As tolerance of *Sophora davidii*, which was closely related to AMF, improving their growth, gas exchange, and chlorophyll fluorescence parameter, reactive oxygen species, and antioxidant enzymes while decreasing As accumulation in *S. davidii* tissues (Zhang et al., 2022). The above studies investigated the positive effects of AMF inoculation on As tolerance of host plants by using phenomic approaches. The establishment of AMF symbiosis involves complex signaling events that often involve the recruitment and modification of gene expression patterns in host plants to fulfill the symbiotic requirements, and this process ultimately leads to changes in the expression of mycorrhiza-regulated genes in the host plants (Song et al., 2014). RNA sequencing (RNA-Seq) is an effective method for investigating the transcriptome-wide response of plants and elucidating the underlying molecular mechanisms (Li et al., 2020). This method generates vast annotation information on differentially expressed genes, providing opportunities for function analysis of genes regulated by specific factors (Ma et al., 2018). However, a comprehensive transcriptome analysis, to elucidate the molecular mechanism involved in the symbiotic process of AMF and host plants under As stress, is still not completely understood.

The phytoremediation potential of *Pteris vittata* L. (*P. vittata*), an As hyperaccumulator with strong As accumulation capabilities, demonstrated in various remediation projects in As-contaminated areas of China (Wan et al., 2016; Sun et al., 2023). The inoculation of AMF (*Claroideoglomus etunicatum*) increased the phytoremediation efficiency of *P. vittata* in As-contaminated soils through hyphal uptake, promotion of plant growth, and alteration of rhizosphere environment (Pan et al., 2024). As absorption by mycorrhizal hyphae can be transported and relayed to mycorrhizae via symbiotic structures, such as arbuscular, accounting for approximately one-third of As in symbionts (Pan et al., 2024). Despite these advancements, challenges such as slow plant growth and low biomass of *P. vittata* hindered the overall phytoremediation efficiency. In recent years, China actively promoted the advancement of soil As pollution technologies, including the intercropping remediation approach that integrated hyperaccumulated plants, microorganisms, and economic crops on As-contaminated farmland (Ministry of Environmental Protection, 2015). This approach aims to enhance phytoremediation outcomes while also promoting sustainable agricultural practices and economic viability. Cotton (*Gossypium* spp.) is a fiber crop with large biomass, a developed root system, and high economic value, and it also shows more excellent tolerance (Bharwana et al., 2014; Li et al., 2020). Xinjiang Autonomous Region is the main cotton producing region in China; in Kuitun Prefecture of Xinjiang, there are 134,700 hectares of cotton planting area, and groundwater is the main source of irrigation water. However, the groundwater in Kuitun is contained with a natural As level of 800 ug/L, which exceeds the set limit of 50 ug/L for drinking water and 100 ug/L for irrigation (Chen et al., 2018). As a non-edible crop, *Gossypium* is in no risk of transferring HM into the food chain of humans; laboratory experiments show that the HM contents in the fiber of *Gossypium* grown in HM-polluted soils are negligible, making it a promising candidate for phytoremediation in HM-contaminated areas (Li et al., 2020). Planting cotton has been considered for potentially remediating HM-polluted soils in southern China (Ma et al., 2017). To gain a deeper understanding of the molecular mechanisms underlying the alleviation of As phytotoxicity by AMF symbiosis in cotton seedlings, a pot experiment was conducted for investigating the effect of *F. mosseae* inoculation on Ca^2+^ flux and concentration in *Gossypium* seedlings under As stress. Subsequently, transcriptomic analysis was used to elucidate the specific molecular mechanism of the Ca^2+^ signaling pathway, which was induced by *F. mosseae* inoculation under As stress. Our findings provide novel insights into their As tolerance mechanism of plants regulated by AMF and also offer a molecular theoretical basis for the ecological restoration function of mycorrhizal plants grown in As-contaminated soil in the future.

## 2 Materials and methods

### 2.1 Plant materials, AMF inoculum, and pot experiment

Seeds of cotton (*Gossypium* spp. cultivar: Dalingmian 69) were sourced from the Institute of Cotton Research, Chinese Academy of Agricultural Sciences. The cotton seeds were sterilized in 75% ethanol for 20 min, followed by deionized water three times. Subsequently, they were germinated in autoclaved wet sand at 25°C for 10 days. The healthy seedlings were selected and transplanted into plastic containers. *Funneliformis mosseae* (BGC XZ02A) was purchased from the Institute of Plant Nutrition and Resources, Beijing Academy of Agricultural and Forestry Sciences, China. *F. mosseae* inoculum was placed 3 cm below in each pot of mycorrhizal treatment, half of the pots received 30 g *F. mosseae* inoculum and the other half received 50 mL suspensions of 30 g of unsterilized *F. mosseae* inoculum filtrated by 10 μm ultrafiltration membrane.

### 2.2 Experimental procedure and design

The seedlings were cultivated in a greenhouse with a temperature of 20–35°C and relative humidity of 60–85% from April to July 2020. Each pot was supplemented with 100 mL of Hoagland’s solution (2.0 mmoL/L) weekly (Hoagland and Arnon, 1950; Liu et al., 2020), and the soil moisture was kept at 75% field capacity with deionized water by regular weighing. According to Risk Control Standard of Soil Pollution in Agricultural Land in China (GB 15618–2018), when the As concentration in farmland soil was higher than 100 mg/kg, it was considered high risk and was forbidden to plant agricultural products. The As^5+^ addition levels (100 mg·kg ^−1^ dry soil) were applied as Na_3_AsO_4_ •12H_2_O solution which was thoroughly mixed with soils, in order to ensure uniform distribution of As in the soil. All *Gossypium* seedlings were harvested 3 months after AMF inoculation.

The design of the present study was set up in a completely randomized block arrangement with two factors: (1) AMF treatments, i.e., *F. mosseae* and a non-AMF inoculated control; (2) two As levels in soils, i.e., 0 and 100 mg kg^ −1^ dry soil, respectively. Thus, this experiment included four treatments: (1) non-inoculated *Gossypium* under non-As stress (CK0), (2) non-inoculated *Gossypium* under As stress (CK100), (3) *F. mosseae*-inoculated *Gossypium* under non-As stress (FM0), and (4) *F. mosseae*-inoculated *Gossypium* under As stress (FM100). Hence, each of the four treatments had three replicates, making 12 pots (one seedling per pot).

### 2.3 Assessment of AMF colonization

Approximately 1 cm long segments of fine capillary root were cleared in 10% KOH (w/v) at 90°C for 60 min and acidified in 1% HCl (v/v) for 5 min. Then, the root samples were stained with 0.05% trypan blue (w/v) at 90°C for 30 min and destained overnight in a lactoglycerol solution (lactic acid, glycerol, and water, 1:1:1) (Phillips and Hayman, 1970). Forty root segments were randomly selected from each root sample, mounted in glycerin on microscope slides with coverslips, and examined at ×100–400 magnification under a microscope (Nikon YS100, Japan). The stained sub-samples were microscopically examined to determine the percentage of root pieces containing arbuscules, vesicles, and hyphae. Total colonization (T%), arbuscular colonization (A%), vesicular colonization (V%), and hyphal colonization (H%) were defined as the percent of mycorrhizal colonization in fine root segments at 15, 30, 45, 60, 75, and 90 days post-inoculation (dpi), respectively. The rates of mycorrhizal colonization were calculated using the method by Giovannetti and Mosse (1980). The percentage of AM fungal colonization was assessed and calculated as follows: Fungal colonization = 100% × (number of intersections with fungal structure/total number of counted intersections) (Ban et al., 2023).

### 2.4 Detection of Ca^2+^ fluxes in *Gossypium* leaves and roots

Ca^2+^ fluxes were measured by using the non-invasive micro-test technique (NMT-YG-100, Younger USA LLC, Amherst, MA, United States). After 3 months of growth, the *Gossypium* root tips (1–2 cm) and the middle of the third leaves were used for measuring steady ion fluxes. Each ion was measured by a specific selective micro-electrode for Ca^2+^. A continuous recording of ion flux was taken for 300 s for each sample. After drying at 75°C for 24 h, *Gossypium* leaves and roots were ground into powder. Ca^2+^ ions were extracted by H_2_O_2_-H_2_SO_4_ using a microwave digestion instrument (Bayue BYWB-40, Changsha, China) and, subsequently, assayed using an atomic absorption spectrophotometer (PerkinElmer PE800, Waltham, MA, USA).

### 2.5 Total RNA extraction, library preparation, and illumina HiSeq sequencing

The total RNA of *Gossypium* entire plant was extracted from 12 *Gossypium* samples using the tTRIzol® Reagent, according to the manufacturer’s protocol (Invitrogen, CA). The concentration and purity of extracted RNA were analyzed using a NanoDrop spectrophotometer ND-2000 (Thermo Fisher Scientific Inc., Waltham, MA, USA). Only high-quality RNA sample was selected for constructing the sequencing library. After purification, end repair, and ligation to sequencing adapters, RNA-seq transcriptome library was prepared by TruSeq RNA Sample Preparation Kit (Illumina Inc., San Diego, USA), according to the manufacturer’s instructions. Finally, libraries generated from the plants were sequenced by using the Illumina NovaSeq 6,000 platform at BIOZERON Co., Ltd (Shanghai, China). The raw reads are available in the NCBI SRA database under the accession number PRJNA1062123 (Release date: 2028-01-06).

### 2.6 *De novo* assembly, functional annotation, and identification of differential expression genes

Before transcript assembly, Cutadapt v2.10 software was employed to cut off adapter sequences (Martin, 2011). To obtain more reliable clean reads, greater than 10% undetermined bases, low-quality reads (Q-value ≤ 20), and poly-N were discarded. Trinity v2.10.0 software was operated for assembling with the filtered data and constructing the unigene set of sequences (Grabherr et al., 2011). Clean data from each sample were mapped back onto the assembly transcripts (mismatch = 2), and according to the mapping results, the read count for each gene was received using RSEM software. The corresponding fragments per kilobase of transcript per million mapped reads (FPKM) were obtained by calculating the number of mapped and filtered reads of each unigene (Trapnell et al., 2010). All unigenes were annotated through BLASTX against the databases NCBI protein nonredundant (NR), Swiss-Prot, Clusters of Orthologous Groups of proteins (COG), Gene Ontology (GO), and Kyoto Encyclopedia of Genes and Genomes (KEGG) at an E-value cutoff of 1e^−5^.

To detect differential expression genes (DEGs) between two different samples, the expression levels of each transcript were measured by the method of reads per kilobase of exon per million mapped reads (RPKM). RSEM online software (http://deweylab.biostatwisc.edu/rsem/) was used to quantify transcript abundances. Gene differential expression analysis was carried out by using R statistical package software EdgeR (http://www.bioconductor.org/packages/2.12/bioc/html/edgeR.html). To determine the transcriptional changes in the two species under As stress, DEGs were identified by comparing the expression levels at CK0 versus Fm0 and CK100 versus Fm100, respectively. To fully determine which genes were specifically modulated during AMF inoculation and/or As stress, transcripts with *p*-value < 0.05 and |log_2_FoldChange| ≥ 2 were identified as DEGs between any of the groups.

### 2.7 Functional enrichment of DEGs and identification of transcription factor

To gain insight into the functional categories of DEGs regulated by As stress and *F. mosseae* inoculation in *Gossypium* seedlings, GO and KEGG enrichment analyses were performed for DEGs in CK0 versus FM0 and CK100 versus FM100 comparisons. These analyses aimed to identify significantly enriched DEGs in GO terms and KEGG metabolic pathways at *P* -value ≤ 0.05 under the whole-transcriptome background by Goatools (https://github.com/tanghaibao/Goatools) and KOBAS (http://kobas.cbi.pku.edu.cn/home.do). TFs were identified with the Plant Transcription Factor Database v5.0 (http://planttfdb.cbi.pku.edu.cn/family).

### 2.8 Co-expression network analysis

A gene co-expression network analysis was conducted for *CBLs* and *CIPKs* in *Gossypium* using the Pearson correlation coefficient (PCC), which was calculated using SPSS 25 software (SPSS Statistics, IBM, United States). All CBL and CIPK gene pairs with PCC of *p* < 0.05 were collected using Cytoscape 3.10.1 software, to create the co-expression networks (Shannon et al., 2003). Similarly, a co-expression network of transcription factors and Ca^2+^ signaling pathway-related genes of *Gossypium* was also performed and displayed by the above method. Only TFs with a sub-network of at least two genes were considered for further analysis.

## 3 Results

### 3.1 Mycorrhizal colonization

**Figure 1:**
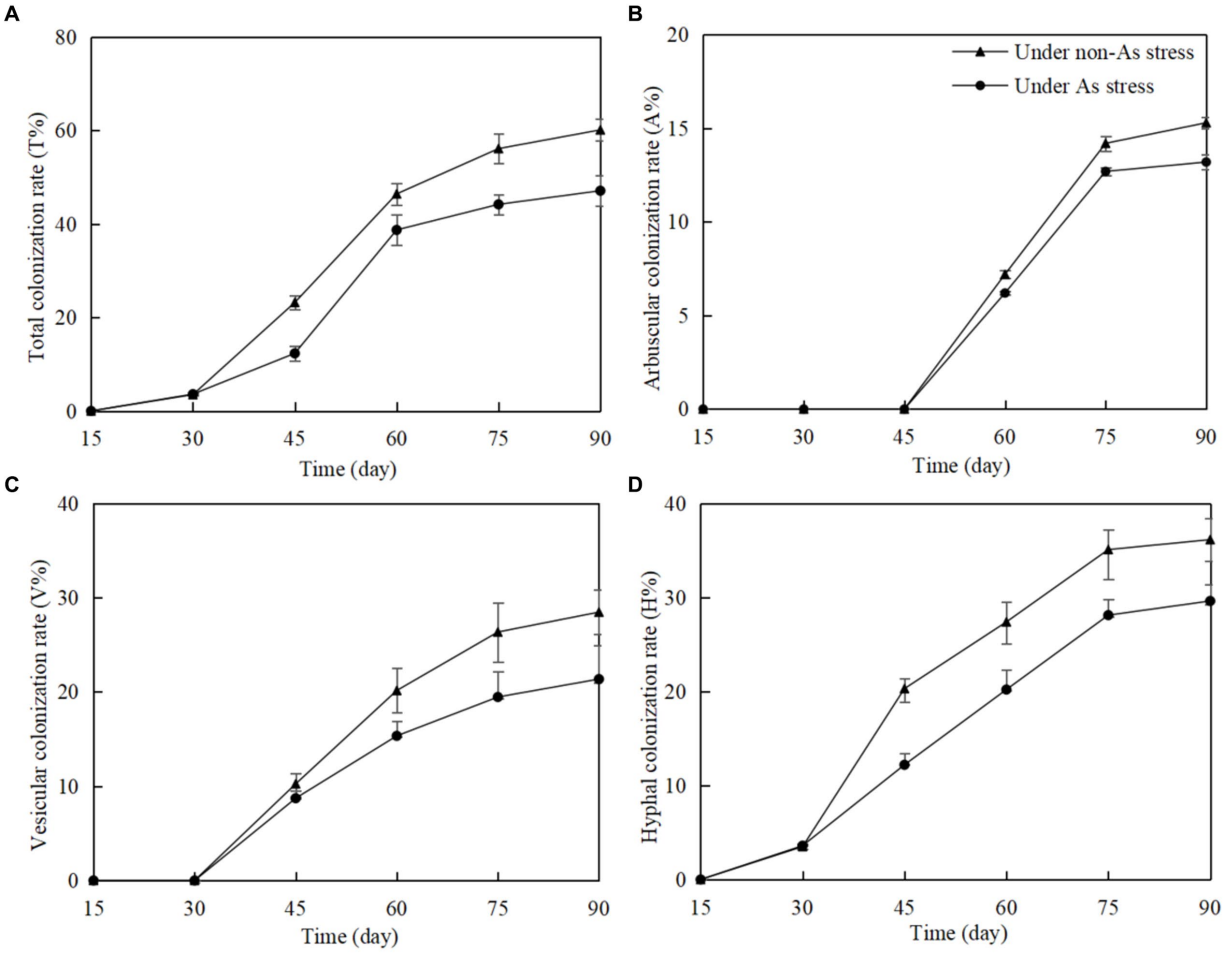
*Funneliformis mosseae* colonization rate in *Gossypium* roots at different growth time under non-As stress and As stress **(A)** total colonization rate; **(B)** arbuscular colonization rate; **(C)** vesicular colonization rate; **(D)** hyphal colonization rate.

In non-inoculated *Gossypium* roots, no structures of arbuscular mycorrhizal fungi (AMF), including hyphae, arbuscules, and vesicles, were found. However, in *F. mosseae*-inoculated roots, total colonization rate (T%), arbuscular colonization rate (A%), vesicular colonization rate (V%), and hyphal colonization rate (H%) increased significantly with time of post-inoculation (Figure 1). As stress in soils had a negative influence on *F. mosseae* colonization in *Gossypium* roots. Rapid T%, V%, and H% increase began on 30 days post-inoculation (dpi), and A% increase began on on 45 dpi. T%, A%, V%, and H% remained stable from 75 dpi. At 90 dpi, under non-As stress, T%, A%, V%, and H% were 57.12, 15.3, 27.12, and 36.50%, respectively. Under As stress, these values were lower at 46.32, 13.2, 20.45, and 28.42%, respectively.

### 3.2 Ca^2+^ Fluxes and contents in *Gossypium* roots and leaves

**Figure 2:**
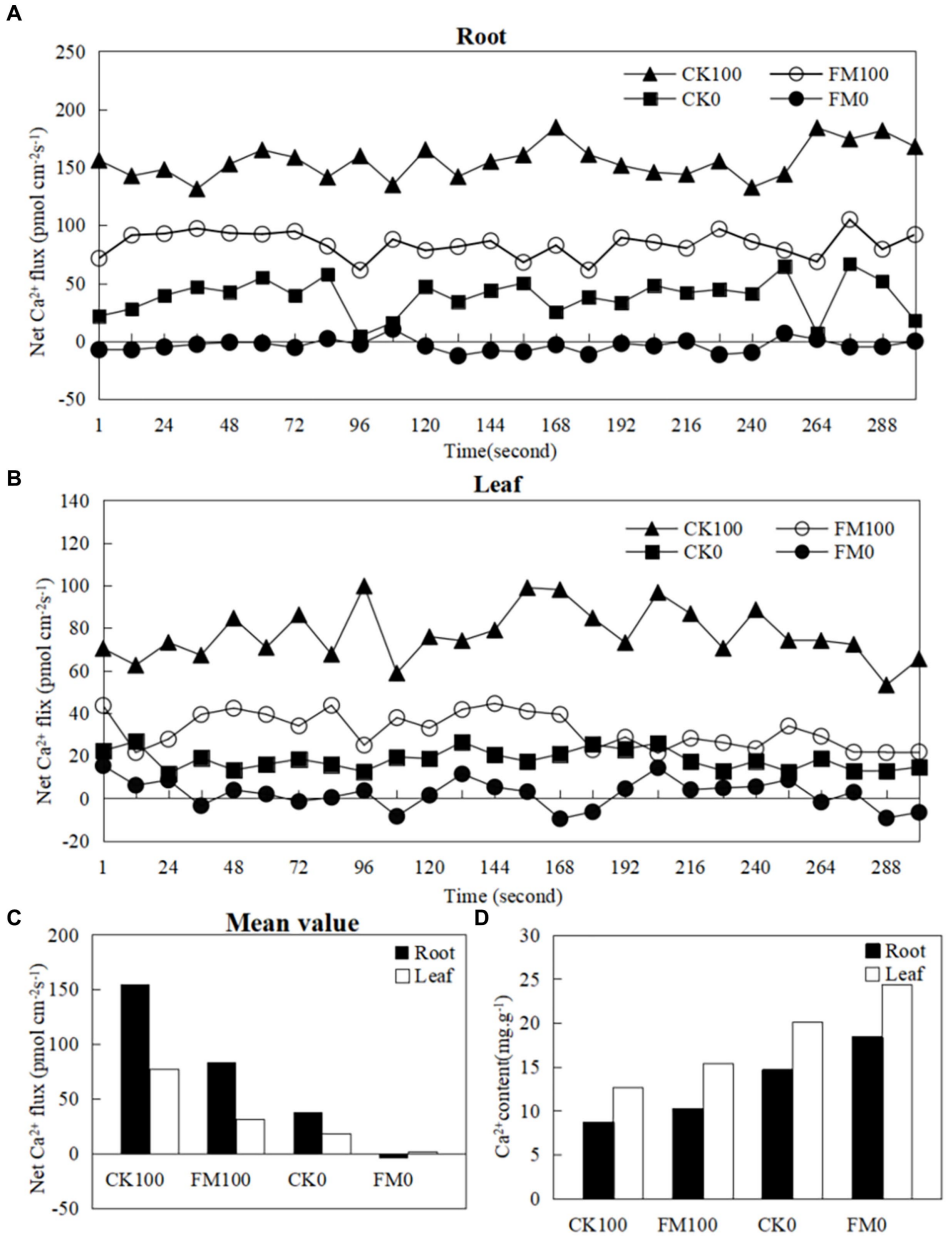
Effects of As stress and *F. mosseae* on the net Ca^2+^ fluxes and Ca^2+^ content in *Gossypium* roots and leaves. The flux rates of Ca^2+^ were measured for 300 s. **(A)** The root of net Ca^2+^ fluxes; **(B)** the leaf of net Ca^2+^ fluxes; **(C)** the mean value of net Ca^2+^ fluxes in *Gossypium* roots and leaves; **(D)** the Ca^2+^ content in *Gossypium* roots and leaves.

Ion kinetics investigations revealed the net fluxes of Ca^2+^ in *Gossypium* roots and leaves (Figure 2). As stress increased the net fluxes of Ca^2+^ in *Gossypium* roots (Figure 2A) and leaves (Figure 2B) under consistent inoculation conditions. However, *F. mosseae* inoculation decreased the net fluxes of Ca^2+^ in *Gossypium* roots and leaves under the same As level in soils. Under non-As stress condition, the mean net rates of Ca^2+^ fluxes in roots and leaves of non-inoculated seedlings were 38.58 and 18.15 pmol/(cm^−2^·s). In contrast, roots and leaves of *F. mosseae*-inoculated *Gossypium* were − 3.18 and 2.26 pmol/(cm^−2^·s), respectively (Figure 2C). Under As stress condition, the mean net rates of Ca^2+^ fluxes were 38.58 and 18.15 pmol/(cm^−2^·s) in the roots and leaves of non-inoculated seedlings and were − 3.18 and 2.26 pmol/(cm^−2^·s) in the roots and leaves of *F. mosseae*-inoculated seedlings, respectively (Figure 2C). The Ca^2+^ contents in *Gossypium* leaves were always higher than that in the roots under the same growth conditions. As stress decreased the the Ca^2+^ contents in *Gossypium* roots and leaves under the same inoculation conditions, but *F. mosseae* inoculation increased the Ca^2+^ contents in roots and leaves under the same As level in soils (Figure 2D).

### 3.3 Transcriptome sequencing and gene annotation

The method of RNA-seq was employed for investigating the transcriptional responses of *F. mosseae*-inoculated *Gossypium* under As stress. A total of 12 cDNA libraries, i.e., CK0, CK100, FM0, and FM100, were constructed and sequenced in triplicates for each treatment. After deleting the low-quality raw reads (Q-value < 20) and trimming adaptor sequences, the percentage of Q30 base was > 92% and GC content was > 43% (Supplementary Table S1A). Clean reads were assembled into 73,746 expressed unigenes. The total number of expressed transcripts, all unigenes, and all transcripts were 124,000, 86,620, and 140,451, respectively (Supplementary Table S1B).

To obtain the annotation information of expressed unigenes and transcripts, BLAST search was used for comparing the assembled sequences with the GO, KEGG, COG, NR, Swiss-Prot, and Pfam databases. The obtained unigenes were functionally annotated with gene function, and there were 65,488, 31,198, 61,825, 82,630, 58,267, and 62,158 sequences annotated to GO, KEGG, COG, NR, Swiss-Prot, and Pfam databases, respectively. The total annotated number of expressed unigenes, expressed transcripts, all unigenes, and all transcripts in all six databases were 70,556, 120,016, 82,677, and 135,694, respectively (Supplementary Table S1B).

### 3.4 Identification of differential expression genes (DEGs) related to *Funneliformis mosseae* inoculation and as stress

**Figure 3:**
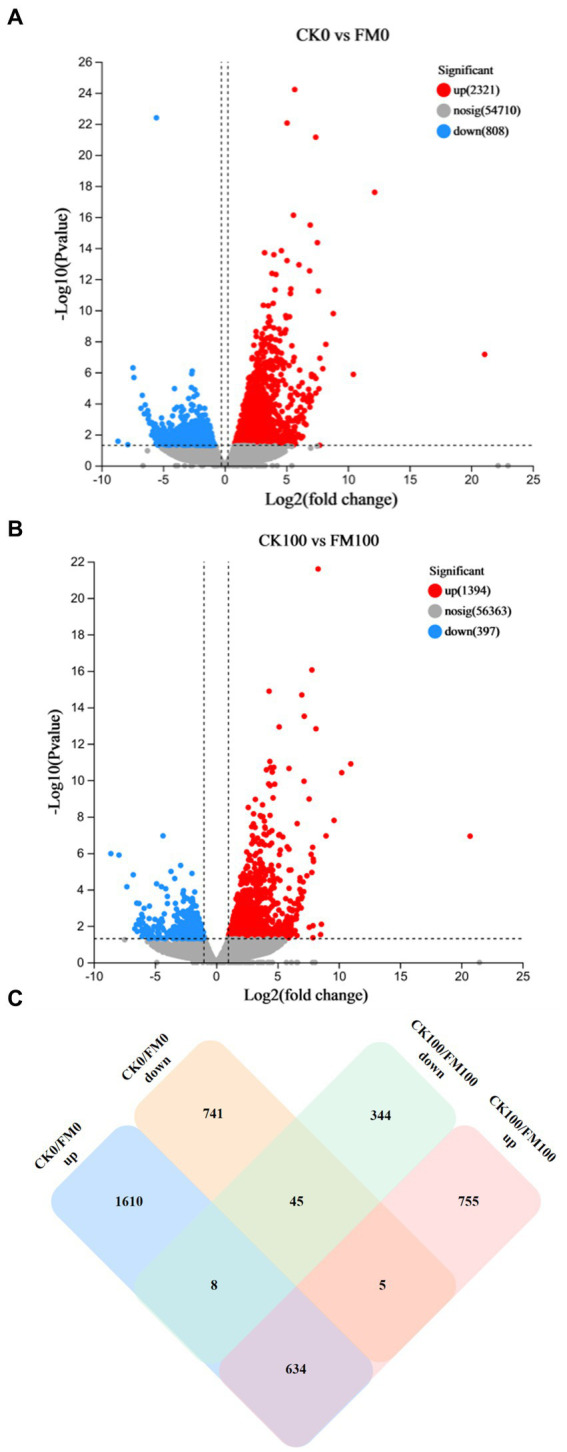
Analysis of upregulated and downregulated DEGs of *Gossypium* in the different comparisons. Volcano plot showed differentially expressed genes **(A,B)**. Green and red dots indicated the significantly downregulated and upregulated genes, respectively. Grey dot indicated the non-significantly different genes. **(C)** Venn diagram.

The identification of DEGs linked to *F. mosseae* inoculation and As stress was achieved through a pairwise comparison of their expression levels. This analysis provided novel insights into the role of AMF symbiosis with *Gossypium* under As stress (Figure 3). Specifically, *F. mosseae* inoculation induced 3,129 DEGs under non-As stress condition; the upregulated and downregulated DEGs were 2,321 and 808, respectively (Figure 3A). When exposed to As stress, *F. mosseae* inoculation induced 1791 DEGs, and the upregulated and downregulated DEGs were 1,394 and 397, respectively (Figure 3B).

A Venn diagram was generated to further explore the relationships among 4,142 DEGs from 4 different comparisons (Figure 3C). The comparison between CK100 and FM100 had 1,394 upregulated DEGs (categorized as 755, 5, and 634) and 397 downregulated DEGs (344, 45, and 8), and these above DEGs induced by *F. mosseae* inoculation may help *Gossypium* seedlings to alleviate As toxicity under As stress. In total, 1,099 DEGs (344 upregulated and 755 downregulated) in CK100 versus FM100 were different from those in CK0 versus FM0 (Figure 3C), and the above DEGs may be specific to *F. mosseae*-inoculated seedlings subjected to As stress. In total, 2,351 DEGs (741 upregulated and 1,610 downregulated) in CK0 versus FM0 comparison were different from those in CK100 versus FM100 comparison, and these DEGs may be specific DEGs in *F. mosseae*-inoculated seedlings under non-As stress conditions. In total, 679 DEGs (45 downregulated and 634 upregulated) were not regulated by As stress to maintain the same original trend, and the above genes are speculated to be key genes, to maintain the symbiotic relationship. Additionally, 13 DEGs (8 and 5) exhibited opposite trends under both As stress and non-As stress, implicating their involvement in regulating both stress responses and symbiotic relationships. Collectively, 1,112 DEGs (comprising 1,099 specific DEGs under As stress and 13 oppositely trending DEGs) were speculated to be specifically involved in the response of *F. mosseae*-inoculated *Gossypium* to As stress.

### 3.5 Functional annotation of DEGs induced by *Funneliformis mosseae* inoculation under as stress

**Figure 4:**
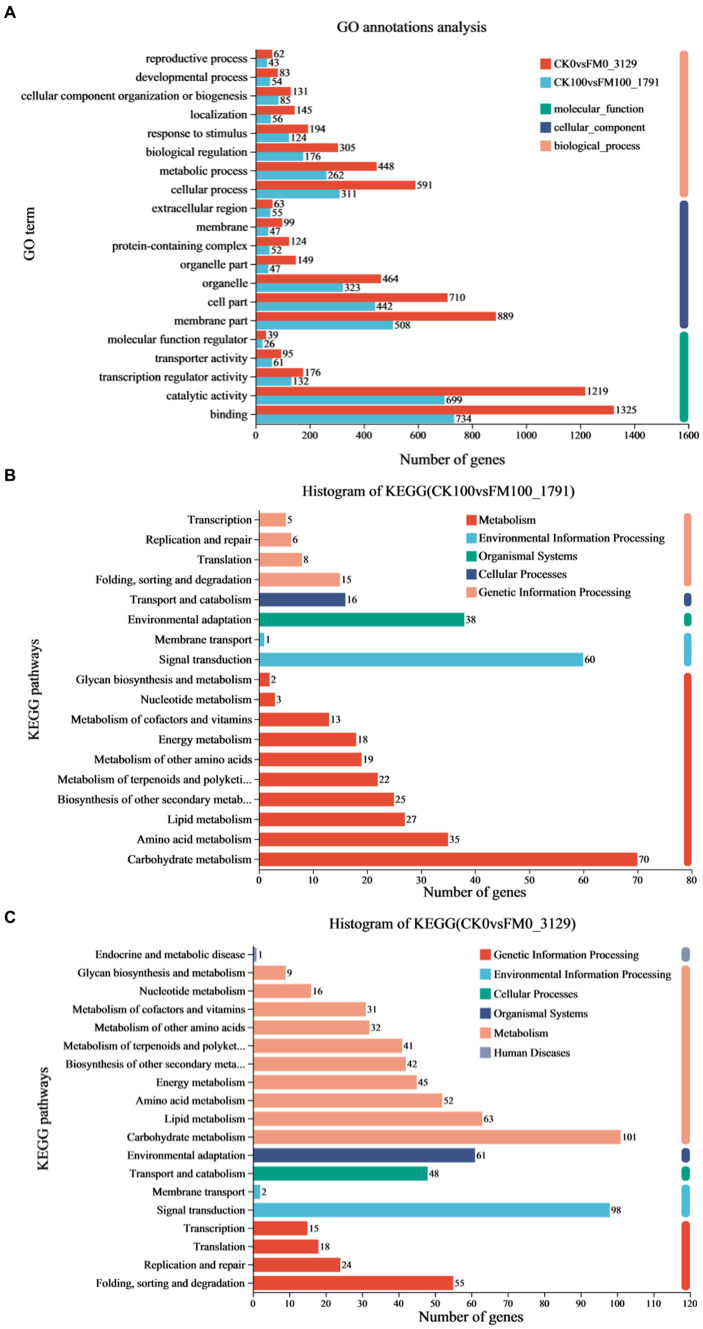
Analysis of functional annotation of DEGs of *Gossypium* induced by *Funneliformis mosseae* inoculation under As stress. **(A)** Significantly enriched biological process, molecular function, and cellular component of GO terms of DEGs in CK100 versus FM100 and CK0 versus FM0 comparison. **(B)** The histogram of KEGG in CK100 versus FM100 comparison. **(C)** The histogram of KEGG in CK0 versus FM0 comparison.

To elucidate the mechanisms underlying the effect of *F. mosseae* inoculation on *Gossypium* seedlings under As stress, 1,791 upregulated and downregulated DEGs in the CK100 versus FM100 comparison were picked for GO and KEGG annotation evaluation (Figure 4). Out of these 1,791 DEGs, 1,441 were successfully categorized into at least one GO term, to characterize the biological processes (BP), cellular components (CC), and molecular function (MF) groups (Figure 4A). Within the BP group, genes were mainly annotated with ‘cellular process’ (311), ‘metabolic process’ (262), ‘biological regulation’ (176), and ‘response to stimulus’ (124). Within the CC group, ‘membrane part’ (508), ‘cell part’ (442), and ‘organelle’ (323) were the most represented. Within the MF group, the ‘binding’ (734), ‘catalytic activity’ (699), and ‘transcription regulator activity’ (132) were predominantly matched terms. Furthermore, the annotation analysis of the KEGG pathways revealed that 383 of 1,791 DEGs were subdivided into at least one metabolic pathway categories: metabolism (M), environmental information processing (EIP), organismal systems (OS), cellular processes (CP), and genetic information processing (GIP) groups (Figure 4B). DEGs mostly annotated with ‘folding, sorting, and degradation’ (15) in the GIP group, ‘transport and catabolism’ (16) in the CP group, ‘environmental adaptation’ (38) in the OS group, and ‘signal transduction’ (67) in the EIP group. In the M group, DEGs mainly annotated with ‘carbohydrate metabolism’ (70), ‘amino acid metabolism’ (35), ‘lipid metabolism’ (27), ‘biosynthesis of other secondary metabolites’ (25), ‘metabolism of terpenoids and polyketides’ (22), ‘metabolism of other amino acids’ (19), and ‘energy metabolism’ (18).

A comprehensive functional analysis of 3,129 differentially expressed genes (DEGs) was conducted in the CK0 versus FM0 comparison, utilizing GO and KEGG annotations. Among these DEGs, 2,521 were successfully categorized into at least one GO term across three functional categories to describe their BP, CC, and MF (Figure 4A). Within the BP group, DEGs mainly annotated with ‘cellular process’ (591), ‘metabolic process’ (448), ‘biological regulation’ (305), ‘response to stimulus’ (194), ‘localization’ (145), and ‘cellular component organization or biogenesis’ (131). Within the CC group, the dominant subcategories were ‘membrane part’ (889), ‘cell part’ (710), ‘organelle’ (464), ‘organelle part’ (149), and ‘protein-containing complex’ (124). Within the MF group, the ‘binding’ (1325), ‘catalytic activity’ (1219), and ‘transcription regulator activity’ (176) were predominantly matched terms. The results of KEGG analysis indicated that 754 of 3,129 DEGs in the CK0 versus FM0 comparison were subdivided into at least one metabolic pathway category: M, EIP, OS, CP, GIP, and Human disease (H) groups (Figure 4C). The M group, ‘carbohydrate metabolism’ (14) and ‘lipid metabolism’ (10), the OS group with ‘environmental adaptation’ (61), the CP group with ‘transport and catabolism’ (48), and the EIP group with ‘signal transduction’ were the most represented (98). Within the GIP group, the dominant subcategories were ‘folding, sorting, and degradation’ (55), ‘replication and repair’ (24), ‘translation’ (18), and ‘transcription’ (15).

### 3.6 Functional enrichment of DEGs induced by *Funneliformis mosseae* inoculation under as stress

**Figure 5:**
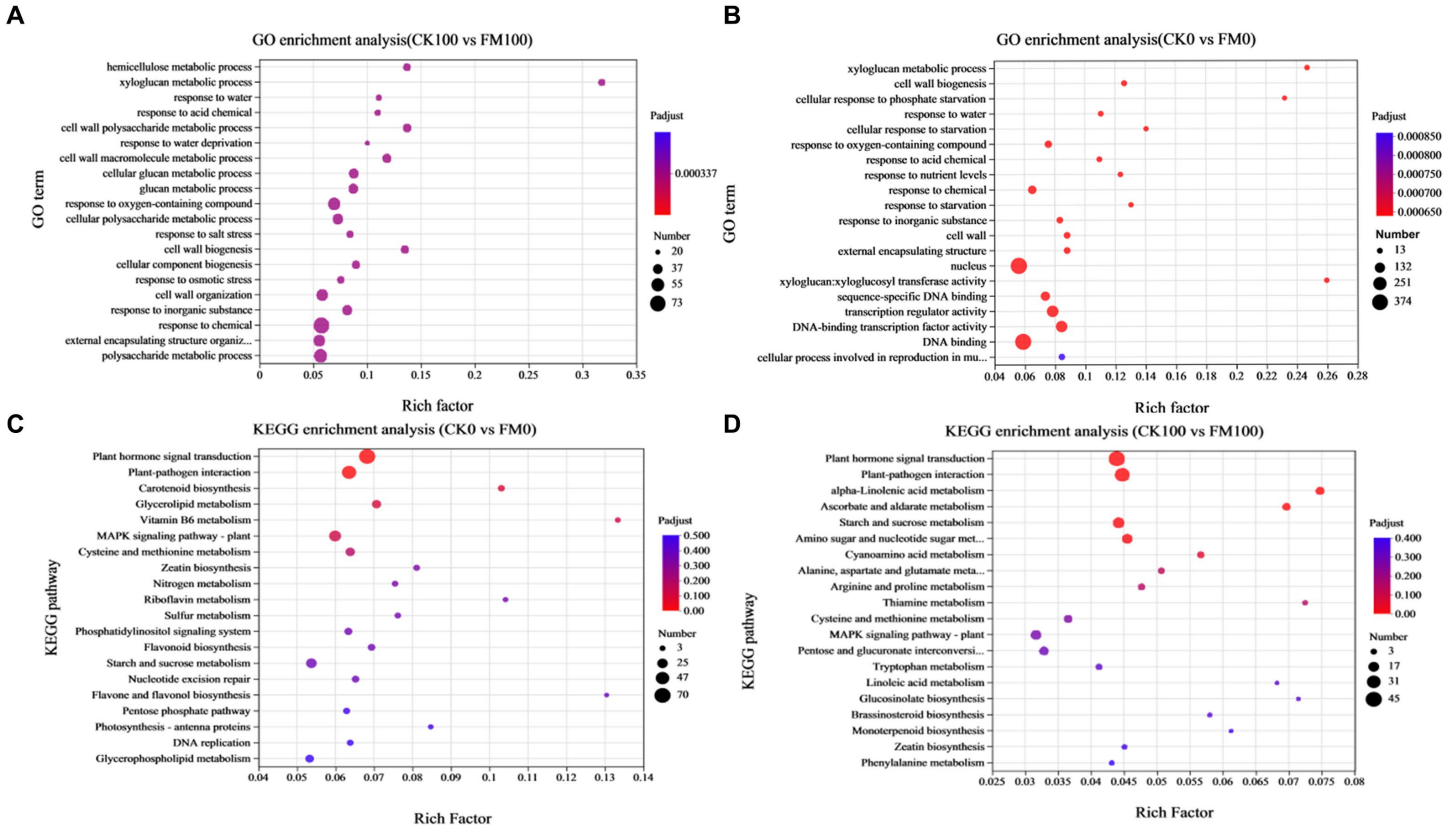
Enrichment analysis of DEGs of *Gossypium* induced by *Funneliformis mosseae* inoculation under As stress. **(A)** The GO enrichment bubble plot of DEGs in CK0 versus FM0. **(B)** The GO enrichment bubble plot of DEGs in CK100 versus FM100. **(C)** The KEGG pathway enrichment bubble plot of DEGs in CK0 versus FM0. **(D)** The KEGG enrichment bubble plot of DEGs in CK100 versus FM100.

GO enrichment analysis was used for elucidating the functions of DEGs, which can be classified under biological process, cellular component, and molecular function (Figure 5). The top 20 significantly enriched GO terms for DEGs (*p*-value ≤ 0.05) are shown in Figure 5. In the CK0 versus FM0 comparison, the most significantly enriched GO terms were ‘xyloglucan: xyloglucosyl transferase activity’, ‘xyloglucan metabolic process’, and ‘cell wall biogenesis’. A larger number of enriched GO terms were ‘membrane-bounded organelle’ (438) and ‘intracellular membrane-bounded organelle’ (431) (Figure 5A). In contrast, for the CK100 versus FM100 comparison, the most significantly enriched GO terms were ‘hemicellulose metabolic process’, ‘xyloglucan metabolic process’, and ‘glucosyltransferase activity’, and a larger number of enriched GO terms were ‘molecular function’ (1186), ‘organelle’ (323), and ‘intracellular organelle’ (322) (Figure 5B).

According to the enrichment results of KEGG pathway analysis, 1,143 (CK0 vs. FM0) and 612 (CK100 vs. FM100) exclusive DEGs were enriched, and the top 20 significantly enriched KEGG pathways for DEGs (*p*-value ≤ 0.05) are shown in Figure 5, which are divided into six KEGG pathways of the first category. For the CK0 versus FM0 comparison, the top pathway of the first category involving the largest number of DEGs was ‘plant hormone signal transduction’ (70), ‘plant–pathogen interaction’ (54), and ‘MAPK signaling pathway-plant’ (36) (Figure 5C), and the most significant enriched pathways of first category were ‘vitamin B6 metabolism’, ‘flavone and flavonol biosynthesis’, and ‘riboflavin metabolism’. For the CK100 versus FM100 comparison, the pathways of the first category involving the largest number of DEGs were ‘plant hormone signal transduction’ (45), ‘plant–pathogen interaction’ (38), and ‘starch and sucrose metabolism’ (23), and the most significant enriched pathways of the first category were ‘alpha-linolenic acid metabolism’, ‘thiamine metabolism’, and ‘glucosinolate biosynthesis’ (Figure 5D).

### 3.7 Expression patterns of DEGs involved in ABC transport protein, calcineurin B-like (CBL)-interacting protein kinase (CIPK) protein, and Ca^2+^-transporting ATPase (CA) regulated by *Funneliformis mosseae* inoculation under as stress

**Figure 6:**
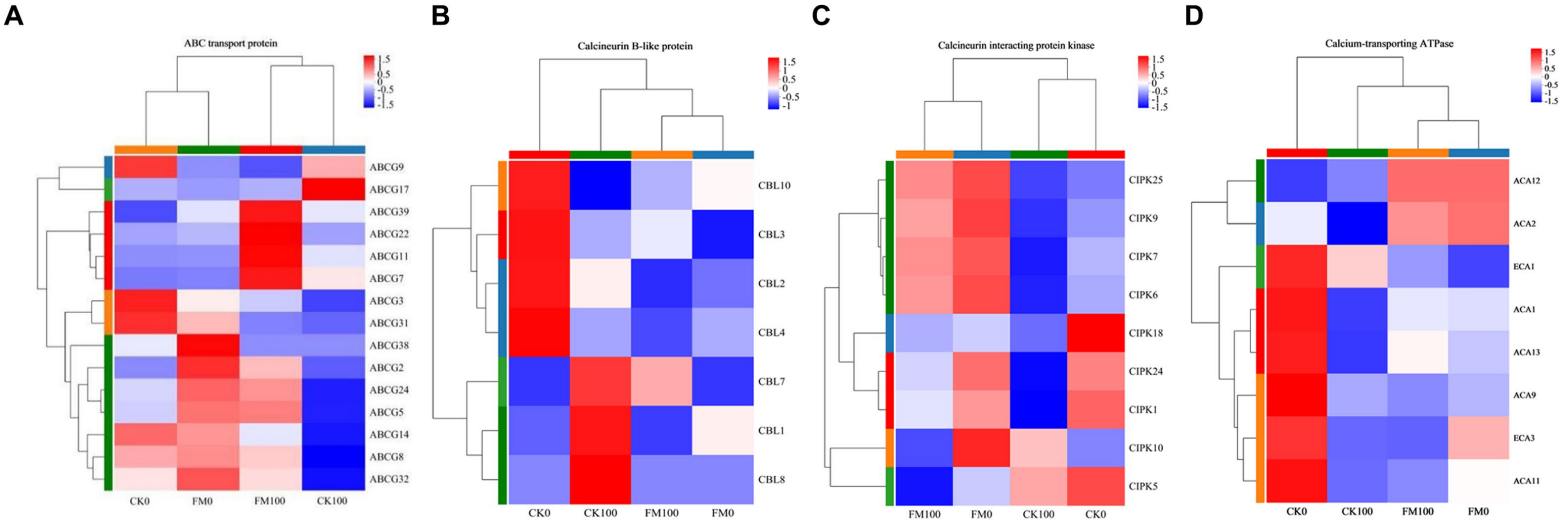
Heatmap of the expression patterns of DEGs of *Gossypium* involved in ABC transport protein, calcineurin B-like protein, calcineurin interacting protein kinase, and calcium-transporting ATPase protein regulated by AMF inoculation under As stress. Each column represents a sample, each row represents a gene, and the colors in the graph represent the expression level of the gene after standardization in each sample.

To further elucidate the molecular mechanisms by which AMF mitigate the adverse effects of As stress, we examined the DEG expression patterns associated with Ca^2+^ signaling pathway genes, such as ABC, CBL, CIPK, and CA. Cluster analysis of gene sets found that 15 *ABC* genes were expressed in *Gossypium* seedlings, and these genes showed differential expression patterns (Figure 6A). *F. mosseae* inoculation downregulated the expression of *ABCG_3_*, *ABCG_9_*, *ABCG_14_*, *ABCG_17_*, and *ABCG_31_*, and upregulated the expression of 10 *ABC* genes (*ABCG_2_*, *ABCG_5_*, *ABCG_7_*, *ABCG_8_*, *ABCG_11_*, *ABCG_22_*^,^ *ABCG24*, *ABCG32*, *ABCG38*, and *ABCG39*) under non-As stress. Under As stress, *F. mosseae* inoculation also triggered the expression of 12 *ABC* genes (*ABCG2*, *ABCG3*, *ABCG5*, *ABCG7*, *ABCG8*, *ABCG11*, *ABCG14*, *ABCG22*, *ABCG24*, *ABCG31*, *ABCG32*, and *ABCG39*) and repressed the expression of *ABCG9* and *ABCG17*. As stress downregulated the expression of 11 *ABC* genes (*ABCG2*, *ABCG3*, *ABCG5*, *ABCG8*, *ABCG9*, *ABCG14*, *ABCG22*, *ABCG24*, *ABCG31*, *ABCG32*, and *ABCG38*) and promoted *ABCG7*, *ABCG11*, *ABCG17*, and *ABCG39* under non-inoculaion condition. It also induced the expression of *ABCG7*, *ABCG11*, *ABCG17*, *ABCG22*, and *ABCG39* and repressed the expression of 10 *ABC* genes (*ABCG2*, *ABCG3*, *ABCG5*, *ABCG8*, *ABCG9*, *ABCG14*, *ABCG24*, *ABCG31*, *ABCG32*, and *ABCG38*) under *F. mosseae*-inoculaion condition.

Cluster analysis of gene sets found that seven CBL genes were expressed in *Gossypium* seedlings, and these genes showed differential expression patterns (Figure 6B). *F. mosseae* inoculation downregulated the expression of *CBL2*, *CBL3*, *CBL4*, and *CBL10* and upregulated the *CBL1* expression under non-As stress. *F. mosseae* inoculation also induced the expression of *CBL3* and *CBL10* and repressed the expression of *CBL1*, *CBL2*, *CBL4*, *CBL7*, and *CBL8* under As stress. As stress downregulated the expression of *CBL2*, *CBL3*, *CBL4*, and *CBL10* and promoted *CBL1*, *CBL7*, and *CBL8* under non-inoculaion condition. Under *F. mosseae*-inoculaion condition, As stress also induced the expression of *CBL3* and *CBL7* and repressed the expression of *CBL1*, *CBL2*, *CBL4*, and *CBL10*.

The nine CIPK genes of *Gossypium* were found to show differential expression patterns by cluster analysis of gene sets (Figure 6C). Under non-As stress, *F. mosseae* inoculation downregulated the expression of *CIPK1*, *CIPK5*, and *CIPK18* and upregulated the expression of *CIPK6*, *CIPK7*, *CIPK9*, *CIPK10*, *CIPK24*, and *CIPK25*. Under As stress, *F. mosseae* inoculation also triggered the expression of *CIPK1*, *CIPK6*, *CIPK7*, *CIPK9*, *CIPK18*, *CIPK24*, *and CIPK25* and repressed the expression of *CIPK5* and *CIPK10.* Under non-inoculaion condition, As stress downregulated the expression of *CIPK1*, *CIPK5*, *CIPK6*, *CIPK7*, *CIPK9*, *CIPK18*, *CIPK24*, and *CIPK25* and promoted *CIPK10.* It also induced the expression of *CIPK1*, *CIPK5*, *CIPK6*, *CIPK7*, *CIPK9*, *CIPK10*, *CIPK18*, *CIPK24*, and *CIPK25* under *F. mosseae*-inoculaion condition.

Cluster analysis of gene sets found that eight calcium-transporting ATPase genes were expressed in *Gossypium*, and these genes showed differential expression patterns (Figure 6D). Under non-As stress, *F. mosseae* inoculation downregulated the expression of *ECA1*, *ECA3*, *ACA1*, *ACA9*, *ACA11*, and *ACA13* and upregulated the expression of *ACA2* and *ACA12*. Under As stress, *F. mosseae* inoculation also triggered the expression of *ACA1*, *ACA2*, *ACA11*, *ACA12*, and *ACA13* and repressed the expression of *ECA1*, *ECA3*, and *ACA9*. As stress downregulated the expression of *ECA1*, *ECA3*, *ACA1*, *ACA2*, *ACA9*, *ACA11*, and *ACA13* and upregulated *ACA12* under non-inoculaion condition. It also increased the expression of *ECA1*, *ACA1*, and *ACA13* and repressed the expression of *ECA3*, *ACA2*, *ACA9*, and *ACA11* under *F. mosseae*-inoculaion condition.

**Figure 7:**
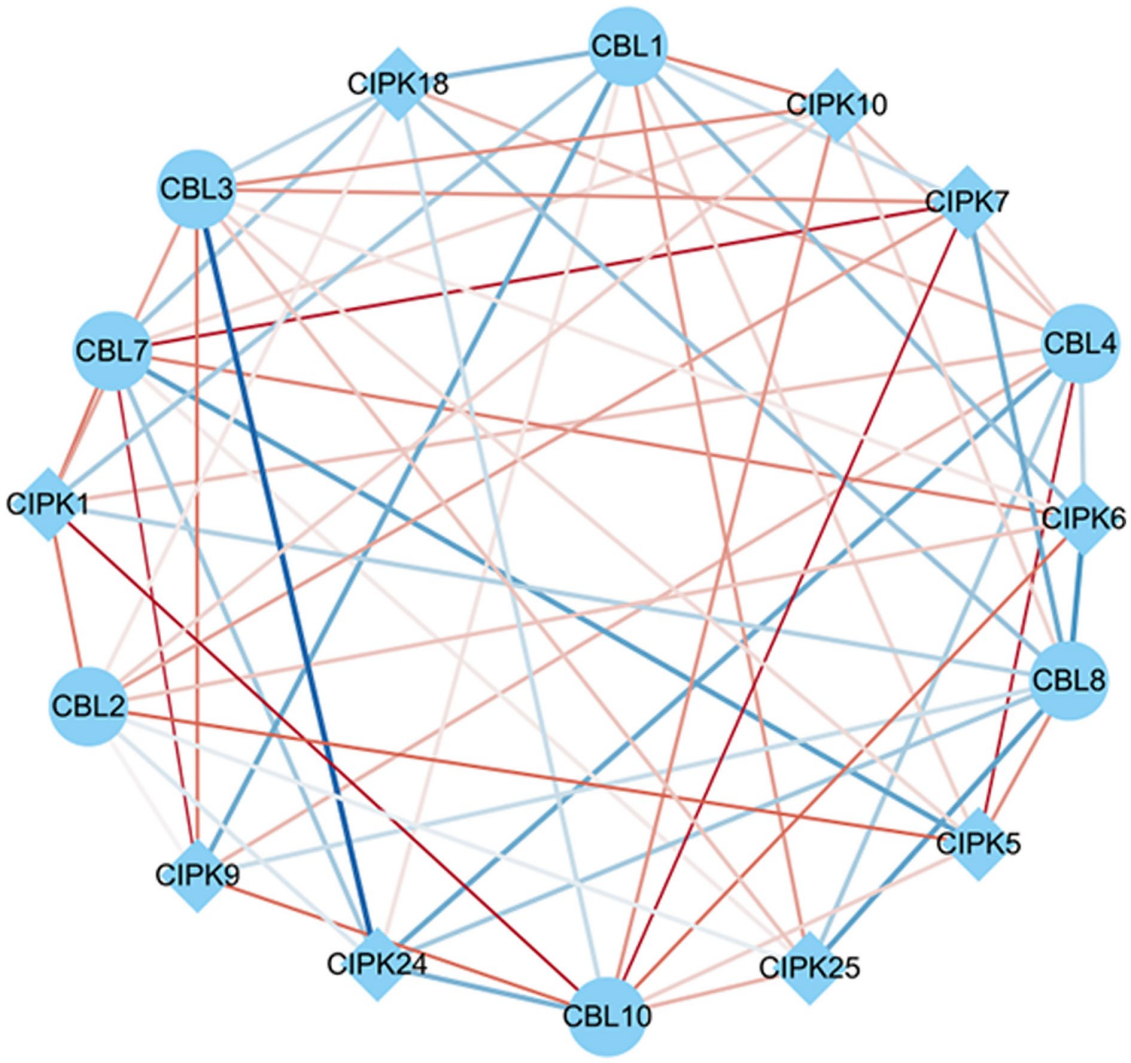
Co-expression networks of calcineurin B-like proteins (CBLs) and calcineurin B-like protein interacting protein kinases (CIPKs) in response to *Funneliformis mosseae* inoculation under As stress. The co-expression networks were established based on the Pearson correlation coefficients (PCC) of the CBL and CIPK pairs. The different line sizes indicate different relevance levels of co-expression gene pairs. Positive correlations are indicated with red lines (PCC ≥ 0.6), whereas blue lines indicate a negative correlations (PCC ≤ −0.6).

Co-expression analysis identified gene pairs of CBL-CIPK signal transduction, responding to As stress and *F. mosseae*-inoculaion. Within this network, 63 regulatory edges represented 63 co-expression gene pairs of *CBLs*-*CIPKs* which are linked by their PCCs. In terms of relatedness, 6 out of 63 had positive and significant correlations. *CBL7* and *CBL10* showed the largest association with *CIPKs*. Among these, *CBL2*-*CIPK5*, *CBL4*-*CIPK5*, *CBL7*-*CIPK7*, *CBL7*-*CIPK9*, *CBL10*-*CIPK1*, and *CBL10*-*CIPK7* had significant positive correlations in response to both As stress and *F. mosseae*-inoculaion (Figure 7).

### 3.8 Identification and co-expression network analysis of transcription factors (TFs)

**Figure 8:**
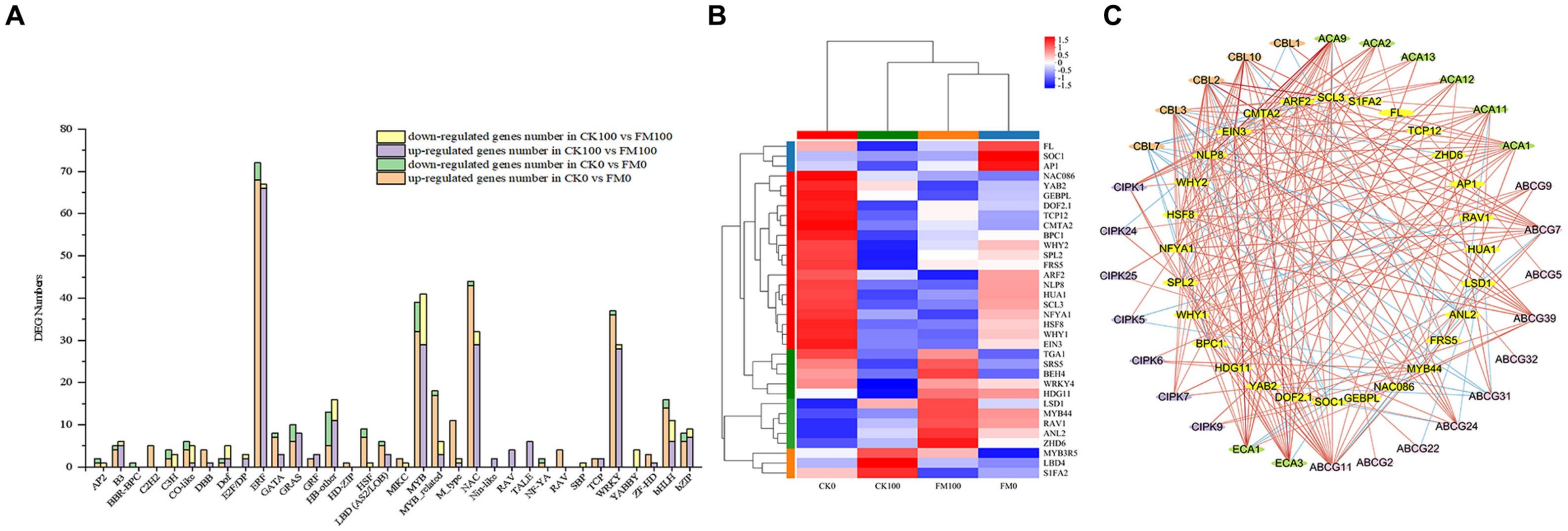
Identification and co-expression network analysis of transcription factors in *Gossypium*. **(A)** Analysis of DEG numbers of transcription factors (TFs) in CK100 versus FM100 and CK0 versuss FM0. X-axis represents TF family, and Y-axis represents DEG numbers. **(B)** Heatmap of the expression patterns of the 34 TFs regulated by AMF inoculation under As stress. **(C)** Co-expression network analyses of 28 TFs regulated by AMF inoculation under As stress. Red and blue lines denote positive correlation and negative correlation, respectively.

To gain insight into the mechanisms by which AMF inoculation alleviates As stress in *Gossypium* seedlings, 336 and 276 DEGs encoding TF family proteins were found to differentially express in CK0 versus FM0 and CK100 versus FM100 comparisons, respectively. The expression of TF genes in *Gossypium* belonged to 34 families, primarily belonging to the TF families of bHLH, HB-other, ERF, MYB, MYB-related, NAC, and WRKY (Figure 8A). The number of upregulated DEGs was much higher than that of downregulated DEGs in both comparisons, respectively. In the CK100 versus FM100 comparison, *F. mosseae* inoculation led to the upregulation of most DEGs in the ERF, MYB, NAC, and WRKY families, while it downregulated DEGs in the MYB and HB-other families. Among these, ERF and MYB had the highest number of upregulated and downregulated DEGs, respectively. Similarly, in CK0 versus FM0 comparison, *F. mosseae* inoculation upregulated DEGs in the ERF, MYB, NAC, WRKY, MYB-related, and bHLH families, while *F. mosseae* inoculation downregulated MYB. ERF and MYB had the highest number of upregulated and downregulated DEGs, respectively.

Characterizing the expression profiles of TFs was a crucial step in transcriptome analyses, 34 TFs were screened and found to significantly express among 4 treatments in this study (Figure 8B). Under As stress, *F. mosseae* inoculation resulted in the upregulation of 23 TFs and downregulation of 11 TFs, respectively. Many of the upregulated TFs, such as *WRKY4*, *MYB44*, and *HSF8*, are known to be involved in abiotic stress response. Downregulated TFs, including *MYB3R5*, *NAC086*, and *ARF2*, were also identified.

The co-expression network analysis was conducted to highlight specific interactions between key TFs and genes related to the Ca^2+^ signaling pathway. Only the TFs with a sub-network of at least two genes were considered for further analysis (6 out of 34 TFs and 9 out of 39 genes). The co-expressed network contained 30 genes associated with the Ca^2+^ signaling pathway and 28 TFs, forming the main network (Figure 8C). The network showed positive correlations between several co-expressed genes and TFs, suggesting TF-inductive regulations. *SCL3* TFs demonstrated the highest number of connections within the main co-expressed network. Additionally, several previously mentioned DEGs with important roles were found to co-express together with specific TFs. For instance, *CBL10* and *ECA1* were co-expressed with *EIN3*, while both TFs *CMAT2* were connected to *ECA3* and *ACA9*. Furthermore, *CBL2*, *CBL2*, *ACA9*, and *ECA3* were co-expressed with *SCL3*.

## 4 Discussion

In our previous studies, *Gossypium* (Cultivar: Dalingmian 69) seedlings were inoculated with *R. intraradices* and *F. mosseae* separately under As stress; the results demonstrated that both AMF strains formed symbiotic relationships with *Gossypium* roots. Furthermore, single *R. intraradices* or *F. mosseae* inoculation individually enhanced the *Gossypium* tolerance to As toxicity by increasing the net photosynthetic rate and light energy utilization efficiency, improving the osmotic regulation and antioxidant capacity, alleviating the As inhibition on root development, and ultimately reducing the arsenic concentration in *Gossypium* seedlings (Liu et al., 2021; Gong et al., 2022). This present study aimed to investigate the transcriptional changes induced by *F. mosseae* inoculation in *Gossypium* seedlings under As stress. Our results revealed that both As stress and *F. mosseae* inoculation induced a large number of changes in gene expression, with 4,998 DEGs identified through transcriptome sequencing.

### 4.1 AMF-induced Ca^2+^ fluxes and contents under as stress

In plants, Ca^2+^ ions serve as a crucial secondary messenger responding to various environmental stimuli, such as heat, salt, cold, drought, and heavy metal stresses (Sanders et al., 1999). Under abiotic stress conditions, plants release Ca^2+^ from both extracellular and intracellular sources into cytoplasm through Ca^2+^ channels, leading to a rapid increase in cytosolic-free Ca^2+^ concentration (Zhu et al., 2020). Ca^2+^ fluxes play a pivotal role in the early defense responses of plants, exhibiting complex spatiotemporal patterns (Kurusu et al., 2010). Positive values of Ca^2+^ fluxes indicate an outflow, while negative values suggested an inflow (Zhu et al., 2020). In this study, the Ca^2+^ fluxes in *Gossypium* leaves and roots were investigated by using non-invasive micro-test technique; As stress increased the net fluxes of Ca^2+^ in both leaves and roots under the same inoculation condition. However, *F. mosseae* inoculation significantly decreased the net fluxes of Ca^2+^ in *Gossypium* leaves and roots exposed to the same As level. This indicated that *F. mosseae* inoculation reduced the Ca^2+^ outflow and increased the Ca^2+^ inflow in *Gossypium* seedling compared with non-inoculated seedling. The increased Ca^2+^ inflow activated Ca^2+^ −binding proteins, upregulated the genes of Na^+^/H^+^ antiporter, and eventually improved the stress resistance of plants (Jiang et al., 2019). Similarly, ectomycorrhizal *Paxillus involutus-*inoculated roots retained higher Ca^2+^ influx than non-inoculated *Populus* × *canescens* roots under cadmium stress. The enrichment of Ca^2+^ in ectomycorrhizal roots was associated with the high capacity of *P. involutus* fungal hyphae for the uptake of Ca^2+^ (Zhang et al., 2017). In our study, As stress decreased Ca^2+^ contents in *Gossypium* roots and leaves under the same inoculation conditions. However, *F. mosseae* inoculation increased Ca^2+^ contents in *Gossypium* roots and leaves exposed to the same As level in soil. Wang et al. (2022) also reported that *R. irregularis* inoculation increased Ca^2+^ contents, reduced the Na^+^/Ca^2+^ ratio, and maintained ion balance in *Casuarina glauca* roots under NaCl stress.

### 4.2 Functional annotation and enrichment of DEGs induced by *Funneliformis mosseae* inoculation under as stress

The establishment of symbiosis between AMF and host plants often led to transcriptional changes in host plants, conferring multiple benefits to plant growth, development, and stress tolerance (Bucher et al., 2014). Annotating and enriching DEGs identified in CK0 versus FM0 and CK100 versus FM100 comparisons can elucidate the similarities and differences of transcriptome expression between non-inoculated and *F. mosseae*-inoculated *Gossypium* seedlings under As stress. Our analysis revealed that both comparisons were significantly enriched in 353 and 90 GO terms, respectively, which indicated that *F. mosseae* inoculation has a broad effect on the gene expression of *Gossypium* seedlings under both As stress and non-As stress conditions. According to Wang et al. (2022), similar transcriptome analysis in *Casuarina glauca* identified 110 and 119 significantly enriched GO terms in RN0 versus RN600 and RA0 versus RA600 comparisons, respectively (where N represents non-inoculated with AMF, A represents inoculated with *R. irregularis*, 0 represents no NaCl stress, and 600 represents 600 mM NaCl stress). Most GO terms appeared in CK100 versus FM100 and were primarily related to biological processes (such as ‘metabolic process’ and ‘cellular process’), cellular components (such as ‘cell part’, ‘organelle’, and ‘membrane part’), and molecular functions (such as ‘binding’, ‘catalytic activity’, and ‘transcription regulator activity’), which indicated that *F. mosseae* inoculation regulated these key pathways in *Gossypium* seedlings to alleviate As toxicity. In these major pathways, *F. mosseae* inoculation could regulate related DEGs, enabling *Gossypium* seedlings to better cope with As stress.

### 4.3 AMF-induced CBL-CIPK gene expression under as stress

The calcineurin B-like protein (CBL)-interacting protein kinase (CIPK) complex was a primary Ca^2+^ sensor in perceiving a range of signals associated with biotic and abiotic stresses (Shu et al., 2020). Stress-induced Ca^2+^ signals were decoded by many Ca^2+^ sensors, including the CBL-CIPK signaling pathway, and in turn, downstream cascades were activated, finally leading to plant physiological and developmental response (Chen et al., 2011; Aslam et al., 2019). *MeCIPK23* interacted with *MeCBL1* and *MeCBL9* in *Manihot esculenta*; over-expression of three genes improved the plant’s defense against *Xanthomonas axonopodis* pv *manihotis* infection (Yan et al., 2018). Similarly, overexpressing *ScCBL* genes in sugarcane (*Saccharum* spp.) resulted in altered expression of immunity genes in *Nicotiana benthamiana* leaves, bolstering the plant’s resistance to *Ralstonia solanacearum* infection (Su et al., 2020). AMF colonization also downregulated three CIPKs in wheat under higher salt stress (Puccio et al., 2023). In *Citrus sinensis*, eight CsCBLs exhibited differential expression patterns in the roots; AMF colonization induced the expression of *CsCBL4*, *5*, *6*, and *7* and repressed the expression of *CsCBL1*, *2*, *3*, and *8* under both well-watered and drought conditions; furthermore, drought significantly downregulated the expression of *CsCBL8* and upregulated *CsCBL7* under non-AMF condition (Shu et al., 2020). The expression profile of *Ananas comosus AcCBL* and *AcCIPK* genes expressed differentially in different tissues and development stages (Aslam et al., 2019). In our study, 7 CBLs and 9 CIPK genes were identified and characterized the expression in *F. mosseae*-inoculated *Gossypium* under As stress, which indicated that these genes play vital roles in the CBL-CIPK signaling pathway under As stress. Not all 7 *CBL*s and 9 *CIPKs* genes were consistently upregulated or downregulated by *F. mosseae* inoculation or As stress, which may be attributed to the integration of gene regulatory elements, such as cis-elements and trans-acting factors.

### 4.4 AMF-induced Ca^2+^-transporting ATPase gene expression under as stress

Ca^2+^−transporting ATPase play a crucial role in plants by transporting Ca^2+^ across cell membranes against an electrochemical gradient using the energy released by the hydrolysis of ATP molecules, which is one of the important physiological processes of the Ca^2+^ signaling pathways in plants (Geisler et al., 2000; Axelsen and Palmgren, 2001). The Ca^2+^-ATPases participated in all the stages of the plant life cycle including growth, development, immunity, and responses to environmental stress (Kamrul et al., 2013). Bago et al. (1997) found that ATPase activities of plasmalemma, mitochondria, and vacuolae were increased in mycorrhizal roots at the later colonization stage. The relative expression levels of both Ca^2+^−transporting ATPase genes (*CA1* and *CA9*) were analyzed in *F. mosseae-*inoculated cucumber roots under chilling stress by using quantitative real-time PCR; the expression levels of both genes in *F. mosseae*-inoculated treatment were increased by 1.60 and 0.74 folds compared with non-inoculated treatment, respectively, which indicated that Ca^2+^ -ATPase genes played an important role in AMF-mediated chilling tolerance of cucumber (Liu et al., 2014). Similarly, in wheat, moss, soybean, and rice seedlings, two genes of P2B Ca^2+^ATPase were upregulated by AMF inoculation under salt stress (Puccio et al., 2023). The expression of some Ca^2+^−transporting ATPase in blueberry seedlings was upregulated by *F. mosseae* inoculation under high pH conditions (Yang et al., 2020). In this study, As stress downregulated 7 of 8 Ca^2+^ −transporting ATPase genes under non-inoculation condition, but *F. mosseae* inoculation upregulated the 5 of 8 Ca^2+^−transporting ATPase genes under As stress, and these findings suggested that *F. mosseae* inoculation positively impacted the tolerance of *Gossypium* seedlings to arsenic by enhancing the gene expression of Ca^2+^−transporting ATPase and improving Ca^2+^ signaling pathway.

### 4.5 AMF-induced ABC transporter gene expression under as stress

The ATP binding cassette (ABC) transporters constitute the most extensive conserved families of integral membrane proteins in plants and play essential roles in the detoxification of HMs in plants by facilitating the transport of phytochelatin-heavy metal (PC-HM) complexes to the vacuoles (Ban et al., 2023). ABC transporter proteins are a group of transmembrane transport proteins commonly found in prokaryotes and eukaryotes, which have a large and diverse range of functions. In plants, ABC transporters can enhance the host plant’s heavy metal tolerance (Guo et al., 2022). As ions with thiol-rich compounds, such as glutathione or PCs, reduced their mobility and sequestered into the vacuole by the ABC transporters (Zhao et al., 2010). Two ABC transporters (*AtABCC1* and *AtABCC2*) in *Arabidopsis* were proven to improve As (V) tolerance in plants (Song et al., 2010). The reports suggest that ABC transporter genes were predominantly expressed in plant cells containing arbuscules, and expression of the upregulated ABC genes was stimulated during the signal communication between AMF and roots of host plants under HM stress (Shabani et al., 2016; Li et al., 2018b). When *Populus* × *canescens* seedlings were inoculated with ectomycorrhizas *Paxillus Involutus*, cadmium stress led to the downregulation of the *ABCC1* gene in mycorrhizal roots (Ma et al., 2013). However, under CuO-NPs and/or flood stress, the gene expression of ABC transporters in *Phragmites Australis* was upregulated by *F. mosseae* inoculation, which indicated that upregulating gene expression of AMF-regulated ABC transporters played a key role in plant response to abiotic stress (Ban et al., 2023). Similarly, ABC transporter transcripts were accumulated to significantly higher levels in *F. mosseae-*inoculated tall fescue compared with non-mycorrhizal plants under nickel (Ni) stress, and these upregulated ABC genes alleviated Ni-induced stress in tall fescue (Shabani et al., 2016). Zhang et al. (2021) further verified that AMF symbiosis enhanced water and nutrient uptake and homeostasis under salinity stress, which induced the expression of DEG-encoding ABC transporter proteins. In our study, under As stress, 15 genes encoding ABC transport protein were screened from the transcriptome gene set and were found to be significantly induced by *F. mosseae* inoculation, and 12 ABC genes were upregulated by *F. mosseae* inoculation, which indicated that AMF symbiosis positively enhanced As tolerance in *Gossypium* seedlings due to the upregulated ABC gene expression.

### 4.6 Regulation of transcription factors by *Funneliformis mosseae* inoculation under as stress

The transcription factors constituted a category of protein sequences that bound to a specific template chain of DNA, thereby governing the transcription from DNA to mRNA (Du et al., 2016; Wang et al., 2022). Many transcription factors, including NAC, MYB, bHLH, ERF, BBX, and WRKY, played pivotal roles in plant growth and development, and they were implicated in the regulation of certain plant genes related to physiological and biochemical processes under abiotic stresses (Cheng et al., 2018). In this study, *Gossypium* seedlings exhibited expression of TF genes belonging to 34 families, with prominent representation from bHLH, ERF, MYB, MYB-related, NAC, and WRKY families. A large number of TF genes (MYB and NAC) related to cell wall pathways were enriched during inoculation with *F. mosseae* under As stress, which suggests that *F. mosseae* inoculation possibly increased the As tolerance of *Gossypium* seedlings by improving the gene expression of TFs related to the development of cell walls and the degree of lignification. Similar results were also found in *R. irregularis*-inoculated *Asparagus officinalis* and *Casuarina glauca* under NaCl stress (Zhang et al., 2021; Wang et al., 2022). The upregulation of MYB, WRKY, ERF, and bHLH TF genes in plant roots was also considered to be an important trigger of the gene expression, underlying the biotic or abiotic stress response (Corso et al., 2018). In this study, the bHLH transcription factors were mainly downregulated by As stress but upregulated by *F. mosseae* inoculation, which was similar to the study by Zhang et al. (2021) on *R. irregularis-*inoculated *Asparagus officinalis* L. under salt stress. Puccio et al. (2023) identified 17 wheat TFs regulated by AMF inoculation, which included two WRKY70, one NAC68, and two bHLH. Overexpression of WRKY, NAC, and bHLH members was proven to improve abiotic and biotic stress responses in *Oryza sativa* L., *Arabidopsis*, and *Nicotiana benthamiana* (Ouziad et al., 2006; Aroca et al., 2007; Corso et al., 2018). Wang et al. (2009) found that NAC TFs regulated defense responses against necrotrophic fungal and bacterial pathogens in tomato seedlings. The WRKY6 TF repressed the expression of *PHT1:1* gene and simultaneously restricted As-induced transposon activation, which decreased As (V) uptake and increased As tolerance of *Arabidopsis thaliana* (Castrillo et al., 2013).

## 5 Conclusion

In our previous studies, *F. mosseae*-inoculated *Gossypium* seedlings exhibited superior physiological performance under As stress, which was attributed to improved photosynthetic, osmotic regulation, and antioxidant capacity, alleviated the As inhibition on root development, and reduced the As concentration in *Gossypium* seedlings (Liu et al., 2021; Gong et al., 2022). In the present study, the inoculation of *F. mosseae* was found to decrease the net fluxes of Ca^2+^ while increased the Ca^2+^ contents in roots and leaves of *Gossypium* under the same As level in soil. To elucidate molecular mechanism related to the Ca^2+^ signaling pathway on how AM fungi could help to alleviate As toxicity, AMF-inoculated and non-inoculated *Gossypium* seedlings under distinct concentrations of Na_3_AsO_4_ •12H_2_O were subjected to high throughput RNA sequencing, respectively. Based on transcriptome data, we provided a global gene profile of *F. mosseae*-inoculated *Gossypium* seedlings under As stress. Specifically, 199 and 3,129 differentially expressed genes (DEGs) were uniquely regulated by *F. mosseae* inoculation under As stress and non-As stress, respectively. Notably, a significant enrichment of genes related to the Ca^2+^ signaling pathway was observed. Furthermore, transcription factors were identified as potentially crucial players in facilitating the relief of As stress. The enrichment of GO (Gene Ontology) and KEGG (Kyoto Encyclopedia of Genes and Genomes) revealed that *F. mosseae* inoculation under As stress upregulated the expression of several Ca^2+^ signaling pathway genes associated with cellular process, membrane part, and signal transduction, which may contribute to alleviating the As toxicity of *Gossypium* seedlings. Additionally, *F. mosseae* inoculation was found to regulate specific DEGs belonging to the transcription factor families ERF, MYB, NAC, WRKY, MYB, and HB-other, which played important roles in mitigating the detrimental effect of As stress on *Gossypium.* Overall, this study provides valuable insights into the molecular theoretical basis underlying the improvement in As tolerance in host plants induced by AMF inoculation, particularly focusing on the Ca^2+^ signaling pathway.

## Data availability statement

The original contributions presented in the study are included in the article/Supplementary material, further inquiries can be directed to the corresponding author.

## Author contributions

MG: Writing – review & editing, Writing – original draft, Funding acquisition. NB: Writing – original draft, Methodology, Formal analysis. JS: Writing – review & editing, Methodology. YuW: Writing – original draft, Validation. YaW: Writing – review & editing, Data curation. QZ: Writing – review & editing, Funding acquisition.
