# Supplementary material for: Transcriptome analysis of Gossypium reveals the molecular mechanisms of Ca2+ signaling pathway on arsenic tolerance induced by arbuscular mycorrhizal fungi
Source: Front Microbiol. 2024 Mar 25;15:1362296. doi: 10.3389/fmicb.2024.1362296 (PMC11000422; doi:10.3389/fmicb.2024.1362296)
Supplement: Supplementary file 1 [file Table_1.DOCX]

| Sample | Raw reads | Clean reads | Clean reads ratio(%) | Q20% | Q30% | GC% | Total mapped (%) | Uniquely mapped (%) |
| --- | --- | --- | --- | --- | --- | --- | --- | --- |
| CK0_1 | 46238628 | 45687646 | 98.81 | 97.78 | 93.28 | 44.35 | 97.21% | 90.14% |
| CK0_2 | 49622804 | 49285186 | 99.32 | 97.62 | 92.76 | 44.32 | 96.73% | 90.84% |
| CK0_3 | 46897754 | 46536094 | 99.23 | 97.60 | 92.80 | 44.23 | 95.29% | 89.5% |
| CK100_1 | 44266796 | 43436452 | 98.12 | 97.67 | 93.04 | 45.44 | 95.3% | 80.77% |
| CK100_2 | 46668256 | 45813006 | 98.17 | 97.34 | 92.23 | 45.07 | 96.76% | 88.54% |
| CK100_3 | 45016848 | 44706570 | 99.31 | 97.35 | 92.10 | 44.54 | 96.36% | 90.77% |
| FM0_1 | 42101244 | 41849878 | 99.40 | 97.51 | 92.44 | 44.35 | 96.49% | 91.09% |
| FM0_2 | 42090474 | 41378480 | 98.31 | 97.50 | 92.57 | 44.88 | 96.76% | 89.54% |
| FM0_3 | 47047624 | 46478256 | 98.79 | 97.45 | 92.49 | 44.29 | 97.05% | 91.71% |
| FM100_1 | 48598564 | 47481844 | 97.70 | 97.52 | 92.79 | 43.69 | 94.64% | 85.24% |
| FM100_2 | 45445792 | 45140726 | 99.33 | 97.31 | 92.01 | 44.50 | 96.37% | 90.84% |
| FM100_3 | 46604696 | 46136540 | 99.00 | 97.50 | 92.54 | 44.43 | 97.24% | 91.38% |

**Table S1** Analysis of transcriptome sequencing and gene annotation

1. Statistics of transcriptome sequencing

**(b)** Annotation analysis of expressed genes and transcripts

| DB name | Expressed Unigene number（percent） | Expressed Transcript number（percent） | All Unigene number（percent） | All Transcript number（percent） |
| --- | --- | --- | --- | --- |
| GO | 56465(0.7657) | 91962(0.7416) | 65488(0.756) | 103976(0.7403) |
| KEGG | 28160(0.3819) | 51105(0.4121) | 31198(0.3602) | 56019(0.3989) |
| COG | 55433(0.7517) | 96501(0.7782) | 61825(0.7137) | 106016(0.7548) |
| NR | 70519(0.9562) | 119959(0.9674) | 82630(0.9539) | 135626(0.9656) |
| Swiss-Prot | 52110(0.7066) | 90147(0.727) | 58267(0.6727) | 99307(0.7071) |
| Pfam | 54924(0.7448) | 92157(0.7432) | 62158(0.7176) | 102426(0.7293) |
| Total annotation | 70556(0.9567) | 120016(0.9679) | 82677(0.9545) | 135694(0.9661) |
| Total | 73746(1.0) | 124000(1.0) | 86620(1) | 140451(1) |
